# Profiling the Tumor-Infiltrating Lymphocytes in Gastric Cancer Reveals Its Implication in the Prognosis

**DOI:** 10.3390/genes13061017

**Published:** 2022-06-05

**Authors:** Weiqiang Yu, Shuaili Wang, Qiqi Rong, Olugbenga Emmanuel Ajayi, Kongwang Hu, Qingfa Wu

**Affiliations:** 1 Division of Molecular Medicine, CAS Key Laboratory of Innate Immunity and Chronic Disease, Department of Life Sciences and Medicine, University of Science and Technology of China, Hefei 230026, China; yuwq@mail.ustc.edu.cn (W.Y.); wangsl01@mail.ustc.edu.cn (S.W.); rqq234@mail.ustc.edu.cn (Q.R.); ajayi@mail.ustc.edu.cn (O.E.A.); 2 Department of General Surgery, Fuyang Hospital Affiliated to Anhui Medical University, Fuyang 236000, China

**Keywords:** gastric cancer, immunotherapy, tumor-infiltrating lymphocytes, hub-genes, prognosis

## Abstract

Gastric cancer is the fifth most common malignancy and the third leading cause of cancer-related mortality worldwide. Immunotherapy offers promising new treatment options for gastric cancer patients; however, it is only effective in a limited fraction of patients. In this study, we evaluated the composition of 22 tumor-infiltrating lymphocytes (TILs) in TCGA Stomach Adenocarcinoma (STAD) using deconvolution-based method by analyzing the publicly available bulk tumor RNA-seq data. The patients were classified into high-TIL and low-TIL subtypes based on their immune cell profiles and prognosis outputs. The differentially expressed genes (DEGs) between the two subtypes were identified, and GO/KEGG analysis showed that broad immune genes, such as PD-L1 and PD-1, were highly expressed in the high-TIL subtype. A comprehensive protein–protein interaction (PPI) network centered on DEGs was built, and 16 hub genes of the network were further identified. Based on the hub genes, an elastic model with 11 gene signatures (*NKG7*, *GZMB*, *IL2RB*, *CCL5*, *CD8A*, *IDO1*, *MYH1*, *GNLY*, *CXCL11*, *GBP5* and *PRF1*) was developed to predict the high-TIL subtype. In summary, our findings showed that the compositions of TILs within the tumor immune microenvironment of stomach cancer patients are highly heterogeneous, and the profiles of TILs have the potential to be predictive markers of patients’ responses and overall survival outcomes.

## 1. Introduction

Gastric cancer (GC) is the fifth most common malignancy and the third leading cause of cancer-related mortality worldwide [1]. In 2015, the incidence and mortality rate of gastric cancer was second among malignant tumors in China [2]. Immunotherapy for stomach cancer, including checkpoint inhibitors and targeted antibodies, offer promising new treatment options for stomach (gastric) cancer patients [3,4]. Tumors are not merely masses of malignant cells but are also complex ecosystems composed of different types of cells. Among these cells, tumor-infiltrating lymphocytes (TILs) play a central role in tumor control and response to therapy [5]. For instance, cytotoxic CD8^+^ T cells are the primary effectors of anticancer immunity, as they can specifically recognize and kill tumor cells bearing neoantigens (i.e., tumor-specific antigens arisen from the expression of mutated genes) [6]. Immune cells can also exert immunosuppressive functions supporting tumorigenesis and immune evasion, as in the case of regulatory T (T_reg_) cells [7]. With the clinical progress of immunotherapy in multiple solid tumors, anti-programmed cell death 1/ligand 1 (PD-1/PD-L1) checkpoint inhibitors have been utilized in the treatment of many types of cancer [8,9]. However, immunotherapy with immune checkpoint blockers is only effective in a limited fraction of patients [10]. Therefore, the quantification of the different types of tumor-infiltrating immune cells can shed light on the mechanisms underlying the anticancer immune response and might help assess the immunogenic effects of anticancer therapies, ultimately guiding the rational design of combination therapies.

The composition of tumor-infiltrating immune cells can be characterized from bulk tumor RNA-seq data using computational approaches based on a set of immune-specific marker genes or expression signatures. The available computational algorithms for immune infiltration estimation fall into two main categories: marker gene-based and deconvolution-based approaches [11,12,13]. Based on the list of marker genes that are characteristic for a cell type, marker gene-based approaches quantify the cell type by performing a statistical test for enrichment of the marker genes [13]. Deconvolution methods define the problem as mathematical equations that model the gene expression of a tissue sample as the weighted sum of the expression profiles from the cells in the population mix [14]. These two complementary categories of algorithms have demonstrated variable performance advantages in estimating specific immune cell types in different tumors. CIBERSORT is one of widely accepted deconvolution-based approaches, which de-convolutes the expression values of 547 marker genes to estimate the infiltration status of 22 immune cell types in tumor samples based on bulk tumor RNA-seq data [14].

The Cancer Genome Atlas (TCGA) molecularly characterized over 20,000 primary cancer and matched normal samples from 33 cancer types, and these data were publicly available [15]. In this study, the expression profiles of STAD patients from TCGA tumors were analyzed to evaluate 22 immune cells infiltration by CIBERSORT method. Then, the patients were classified into high-TIL (tumor-infiltrating lymphocytes) and low-TIL subtypes based on their immune cell profiles and prognosis outputs. The differentially expressed genes (DEGs) between low-TIL and high-TIL subtypes were identified. GO analysis on DEGs revealed that biological processes, including T cell activation and regulation of T cell activation, were up-regulated in the high-TIL subtype. In line with high immune activity, higher expression levels of both PD-1 and PD-L1 were observed in the high-TIL subtype. Tumor mutation burden (TMB) and mutated gene numbers were also significantly higher in the high-TIL subtype. A comprehensive protein–protein interaction (PPI) network centered on the DEGs was built, and 16 hub genes of the network were further identified. Based on the hub genes, an elastic model with 11 gene signatures (*NKG7*, *GZMB*, *IL2RB*, *CCL5*, *CD8A*, *IDO1*, *MYH1*, *GNLY*, *CXCL11*, *GBP5* and *PRF1*) was developed to predict the high-TIL subtype, which showed potential in stratifying stomach cancer patients for immunotherapy.

## 2. Materials and Methods

### 2.1. Data Accession and Processing

The TCGA-STAD RNA-seq expression data, counts and FPKM (FPKM, Fragments Per Kilobase of transcript per million fragments mapped) were downloaded from UCSC Xena (https://xenabrowser.net) in 1 July 2021, containing a total of 407 samples. Similarly, the latest clinical follow-up information and consistent mutation variants annotation were also obtained from UCSC Xena. The cohort of Asian Cancer Research Group (ACRG) consisted of 300 tumor samples was downloaded as the validation dataset for the prediction model developed in project [16]. Only tumor samples with sufficient survival information, 282 TCGA samples and 295 ACRG samples, were included in the downstream analysis. The transcripts per million (TPM) of each gene was transformed into ln(TPM + 1) value and used in downstream analysis.

### 2.2. Characterization of Types and Abundances of Tumor-Infiltrating Lymphocytes

The CIBERSORT method was used to calculate the proportions of 22 immune cell types infiltrated in each STAD sample based on bulk RNA-seq data. For each sample, the permutation parameter for CIBERSORT analysis was 1000, and only samples with statistical significance (*p* < 0.05) were enrolled. ESTIMATE [17] was used to calculate immune cell infiltration scores of patients in three clusters.

### 2.3. Cluster Analysis

R package ConsensusClusterPlus [18] was used to cluster patients with the support immune abundances provided by CIBERSORT. R package ecdf [19] was used to calculate the ‘Empirical Cumulative Distribution Function’ so as to determine the best classification number.

### 2.4. Identification of Differentially Expressed Genes and Hub Gene Identification

The DESeq2 [20] was used to calculate the differentially expressed genes (DEGs) between the high-TIL and low-TIL subtypes and genes with absolute log2 fold change > 1 (Benjamini–Hochberg adjusted *p*-value < 0.05) were regarded as DEGs. The functional exploration for DEGs was performed using a R package [21] with GO and KEGG analysis. The common DEGs identified by both DEGs and EdgeR [22] were assigned higher weights and submitted to STRING website [23] to retrieve the protein–protein interaction (PPI) network. Visualization and hub genes identification were implemented inside Cytoscape [24] with a common plug cytoHubba [25]. 

### 2.5. Statistical Method

The Wilcoxon signed-rank test [26] was used to compare the infiltrated immune cells between normal and tumor samples, as well as high-TIL and low-TIL subtypes. The Kruskal–Wallis test by ranks (sometimes also called the “one-way ANOVA on ranks”) [27] was used to compare PD-1/PD-L1 expression in three groups. The Chi-Square test was used to examine the differences with categorical variables between high-TIL and low-TIL subtypes. Correlation analysis was performed by a R package corrplot [28]. For survival analysis, the Cox proportional hazard model [29] was used to evaluate the immune cluster on survival. All analyses used in this study were implemented with R software (version 4.0.3) [30].

### 2.6. Construction of Predicting Model Relative to High-TIL

In order to train multivariable statistical models for predicting the high-TIL subtype, a total of 282 TCGA tumor samples with sufficient survival information was used for model development. About 198 (70%) samples were selected randomly for the training dataset and the remaining 84 (30%) samples were selected for the test dataset. Using the R package glmnet [31], the elastic net fitting (α = 0.29, λ = 0.03 and 10-fold cross validation) was implemented to perform a penalized multiple logistic regression on all hub genes simultaneously so as to identify the most powerful predictive genes. Patients with a Prediction-Score larger than 0.276 was regarded as the high-TIL subtype. The 295 ACRG samples with sufficient survival information were used as the validation cohort, and the areas under the curve (AUCs) were used to evaluate the performances of the predicting models.

## 3. Results

### 3.1. Characterization of Tumor-Infiltrating Immune Cells of GC Samples

**Figure 1:**
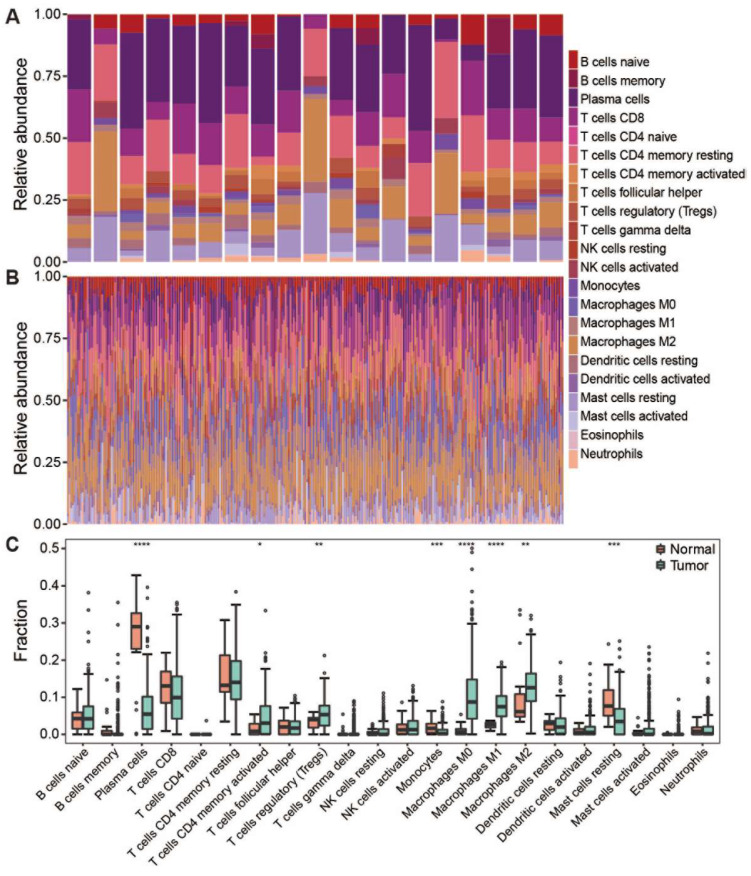
The composition of infiltrating lymphocytes in tumor and normal samples. (**A**) Stacked histogram showed the composition of 22 immune cells in normal samples. (**B**) Stacked histogram showed the composition of 22 immune cells in tumor samples. (**C**) Boxplot showed infiltration of 22 immune cells in normal and tumor samples. Dot means immune cell fraction in each sample. **** *p* < 0.0001, *** *p* < 0.001, ** *p* < 0.01, * *p* < 0.05.

The composition of tumor-infiltrating immune cells can be characterized from bulk tumor RNA-seq data using the CIBERSORT method, a deconvolution-based approach [14]. In this study, we evaluated the composition of 22 immune cell types in TCGA-STAD samples based on the publicly available RNA-seq data. Notably, the infiltrating immune cells have been successfully characterized in 322 out of 407 STAD samples (*p* < 0.05), including 303 tumor samples and 19 normal samples. Highly compositional heterogeneity of the infiltrating immune cells was observed in both tumor and normal samples (Figure 1A,B). The relative proportion of each immune cell type was compared between tumor and normal samples (Figure 1C). Of note, plasma cells, short-lived antibody-producing cell, were significantly dominant in normal samples compared to the tumor samples (*p* < 0.0001). In addition, immune responsive cells such as CD8 T cells, resting Mast cell and Monocytes were also significantly higher in normal samples, suggesting that the infiltration of immune reactive cells into tumors was suppressed in tumor-immune microenvironments. On the contrary, the suppressive immune cells such as Macrophages M0/1/2, regulatory T cells (Tregs), were significantly enriched in the tumor masses (*p* < 0.0001). These results are consistent with the previous report that immune suppression surrounding the tumor is achieved by interfering with antigen-presenting cells and effector T cells [32].

### 3.2. Correlation Analysis for the Immune Cell Types in Tumor Samples

**Figure 2:**
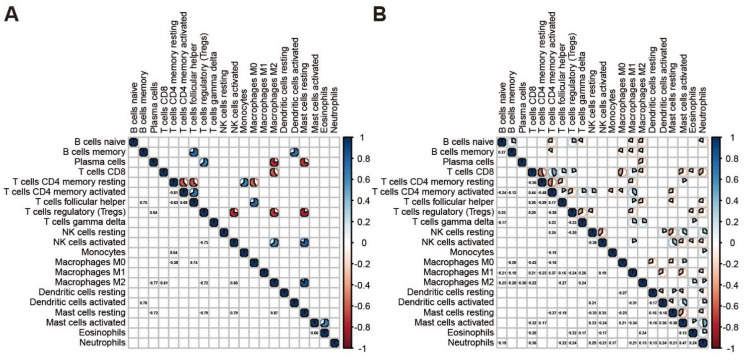
Correlation among immune cells in the tumor and normal samples. (**A**) Corrplot showed the correlation among 22 immune cells in normal samples. (**B**) Corrplot showed the correlation among 22 immune cells in tumor samples.

To characterize the cooperative or antagonistic relationships among the 22 immune cell types in tumors, we carried out a correlation analysis among the 22 immune cell types in tumor and normal samples. In normal samples, significant correlations among fewer cell types were observed although all correlation values are over 0.64 (Figure 2A); in contrast, the correlations among immune cells in tumor tissues were more complicated than that observed in normal tissues (Figure 2B). In tumor samples, we discovered that CD8 T cells are positively related to activated CD4 memory T cells, Follicular helper T cells and Macrophage M1, thus showing that there is cooperation between these cell types (Figure 2B). In contrast, CD8 T cells is negatively related to resting CD4 memory T cells, Macrophage M0 and Neutrophils. Macrophages M0 and M1 show different abilities in the immune response mediated by CD8 T cells. In addition, we noticed that the resting NK cells is positively correlated with activated Mast and negatively correlated with resting Mast. However, activated NK cells are reversed, indicating that NK cells and Mast cells display the opposite trend in the anti-tumor aspects of gastric cancer.

### 3.3. TIL Subtypes and Associated Prognosis of Patients

**Figure 3:**
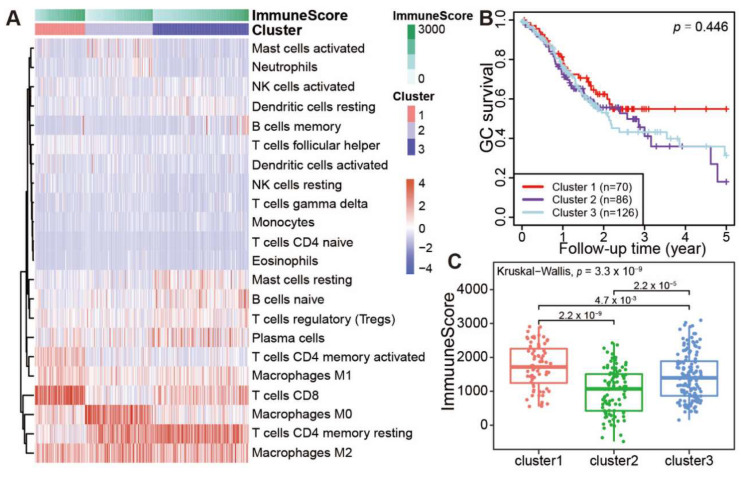
Immune clusters and its associated prognosis of stomach adenocarcinoma (STAD). (**A**) Heatmap showed the immune clusters of STAD. (**B**) Survival status in the three clusters. (**C**) Expression of the immune score in the three clusters.

Based on the immune abundances generated by CIBERSORT, hierarchical cluster analysis was performed, and all tumor samples were grouped into three distinct clusters (Figure 3A). Notably, the three clusters were also well supported by *k*-means cluster analysis based on the immune cell abundances (Figure S1). Obviously, CD8 T cells and activated CD4 memory T cells were highly enriched in the cluster 1. Of note, macrophages M0 cells were exclusively enriched in the cluster 2, in contrast with the general distribution of macrophages M1 and M2 cells in all GC samples. In cluster 2, the mild enrichments of activated Mast cells and Neutrophils were also observed in some samples. Interestingly, the resting CD4 memory T cells is highly enriched in both cluster 2 and cluster 3 samples, in contrast with the scarcity of this cell type in cluster 1. However, for the distributed patterns of resting Mast cells, naïve B cells were obliviously different between clusters 2 and 3. These results suggested that the immune cell patterns among the three clusters are clearly different from each other.

We further investigated whether the clustering of patients is associated with the prognosis in 282 TCGA patients with complete survival information. Notably, the overall survival rate for cluster 1 patients decreased gradually within 2 years after surgery, but it remained stable at 58% after 2.5 years (Figure 3B). However, the survival rate for cluster 2 and cluster 3 patients decreased gradually after surgery, with a 5-year survival rate lower than 30%. As the profiles of infiltrating immune cell types were different among three clusters, the immune cell infiltration scores of three clusters were also calculated by ESTIMATE [17]. The average immune score of each sample in the cluster 1 is significantly higher than cluster 2 or cluster 3 (Figure 3C, cluster 1 vs. cluster 2, *p* = 2.2 × 10^−9^; cluster 1 vs. cluster 3, *p* = 0.0047. Totally, *p* = 3.3 × 10^−9^). These results demonstrated that stronger TILs in patients’ tumor masses indicate better prognosis.

### 3.4. A Broad Elevated Immune Response Identified in Samples with High Prognosis

**Figure 4:**
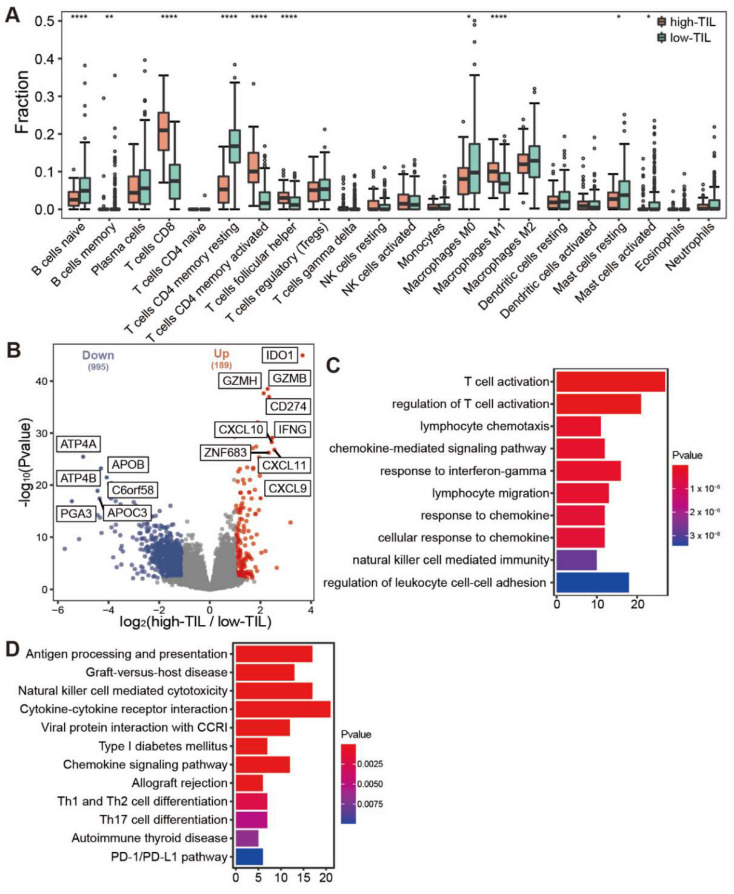
Comparative analysis of immune infiltration and differentially expressed genes between high-TIL and low-TIL subtypes. (**A**) Immune infiltration in high-TIL and low-TIL analyzed by CIBERSORT. (**B**) Differentially expressed genes between high-TIL and low-TIL subtypes. (**C**) Gene Ontology (GO) and (**D**) KEGG enrichment analysis of up-regulated genes in high-TIL subset. **** *p* < 0.0001, ** *p* < 0.01, * *p* < 0.05.

As cluster 2 and cluster 3 had similar prognostic outcomes in comparison to the cluster 1, as well as a lower immune infiltration level, we merged cluster 2 and 3 together, and the group was referred to as low tumor-infiltrating lymphocytes (low-TIL subtype). Correspondingly, cluster 1 with higher infiltrating immune cells and better prognosis is named as high tumor-infiltrating lymphocytes (high-TIL subtype). The relative levels of the infiltration of immune cells were compared between low-TIL and high-TIL subtypes, and the result showed that CD8 T cells, activated CD4 memory T cells, Follicular helper T cells and Macrophage M1 were highly infiltrated in the samples of high-TIL subtype (Figure 4A). 

These samples in high-TIL and low-TIL subtypes originated from 282 individuals of which 181 were male and 101 were female. We found no significant difference in sex (Chi-Square *p* = 0.12) and age at onset (Mann–Whitney U, *p* = 0.35) between the two subtypes (Figure S2A,B). Lauren classification also showed no significant difference between the immune subtypes (Chi-Square *p* = 0.11; Figure S2C). The TCGA project proposed a molecular classification dividing gastric cancer into four subtypes: tumors positive for Epstein–Barr virus (EBV), microsatellite unstable tumors (MSI), gnomically stable tumors (GS) and tumors with chromosomal instability (CIN) [15]. We investigated the proportion of each TCGA subtype in both high-TIL and low-TIL groups and found that either high-TIL or low-TIL subtype is consisted of heterogenous TCGA subtypes that have distinct characteristics (Figure S2D). Notably, the 72.7% EBV subtype belongs to the high-TIL group, in contrast with 42.9% MSI, 11.4% GS and 15.2% CIN.

We further identified the differently expressed genes between the two subtypes. In total, there were 189 up-regulated and 995 down-regulated genes in the high-TIL subtype (Figure 4B). Consistent with the higher infiltrating immune cells, the immune-related genes were highly expressed in the high-TIL subtype, such as *IDO1*, *CD274*, *IFNG*, *CXCL9*, *CXCL10* and *CXCL1*. Gene ontology (GO) analysis revealed that immune-related pathways, such as T cell activation, the regulation of T cell activation, lymphocyte chemotaxis and chemokine-mediated signaling pathway, were enriched in up-regulated genes. KEGG enrichment analysis showed that biological processes, such as antigen processing and presentation, chemokine signaling pathway, Th1 and Th2 cell differentiation, Th17 cell differentiation and PD-1/PD-L1 checkpoint pathway, were enriched for high-TIL subtype (Figure 4C,D). These results indicated that the tumors of high-TIL subtype have a broad elevated immune response. The down-regulated genes with the highest fold change include *ATP4A*, *ATP4B* and *PGA3* (Figure 4B). Of note, the down-regulation of *ATP4A* or *ATP4B* expression has been associated with gastric cancer prognosis [33,34]. A dramatic decrease in *PGA3* may serve as a biomarker for progression of gastric precancerous lesions [35].

**Figure 5:**
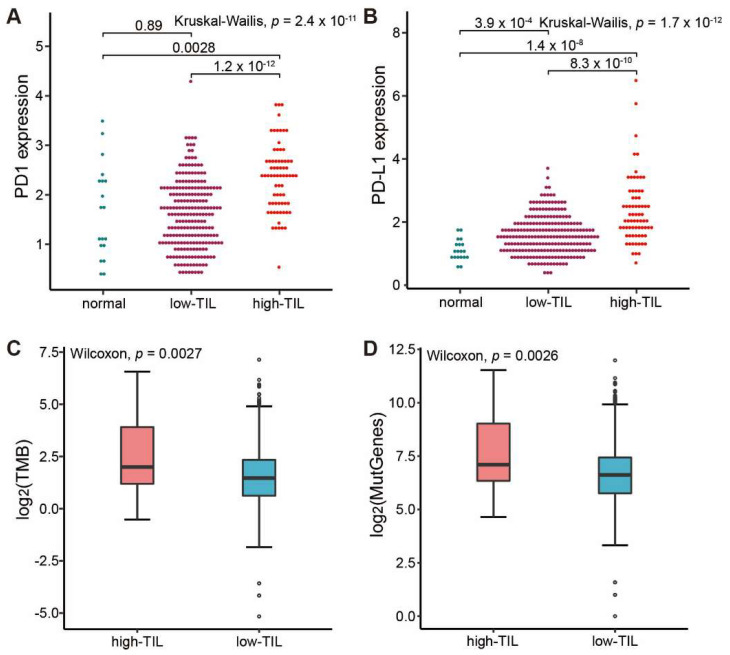
Comparative analysis of ICIs-related biomarkers between high-TIL and low-TIL subtypes. (**A**) PD-1 expression in normal sample, low-TIL and high-TIL samples. (**B**) PD-L1 expression in normal sample, low-TIL and high-TIL samples. (**C**) Tumor mutation load (TMB) in high-TIL and low-TIL samples. (**D**) Mutated gene numbers in high-TIL and low-TIL samples. TMB is calculated by the number of non-synonymous somatic mutations per mega-base in coding regions. The y axis in (**A**,**B**) represents the natural logarithmic value of (TPM + 1).

Of note, the expressions of both *PD-1* and its ligand *PD-L1* were significantly higher in the high-TIL samples (Figure 5A,B), suggesting that the immune cells might be suppressed in tumors. Mutation analysis showed that the tumor mutational burden (TMB) and the counts of mutated genes in these patients with high tumor-infiltrating lymphocytes were also obviously higher than that observed in lower prognosis group (Figure 5C,D). As patients with more mutated genes easily trigger immune responses when treated with immune checkpoint inhibitors (ICIs) [36], our results suggested that the group with higher tumor-infiltrating lymphocytes might benefit from the treatment with immune checkpoint inhibitors (ICIs).

### 3.5. Development of Predicting Model Relative to High-TIL Based on Hub Genes

**Figure 6:**
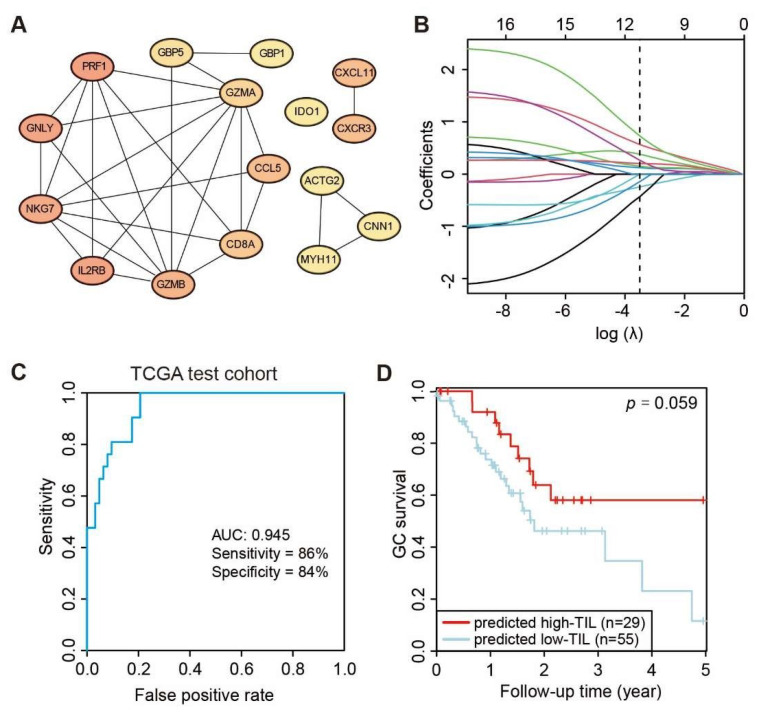

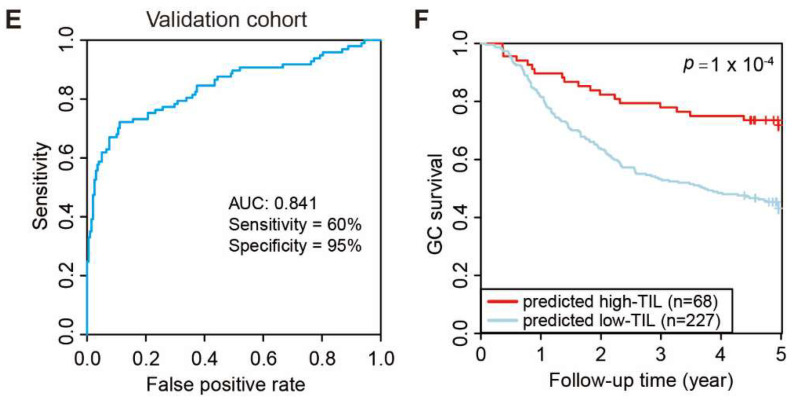
Development of predicting model relative to high-TIL subtype. (**A**) PPI network of 16 hub genes. (**B**) Least absolute shrinkage of the selected genes. (**C**) Receiver operating characteristic (ROC) curves of predictions based on 11 gene expression levels in the TCGA test cohort, *n* = 82. (**D**) Kaplan–Meier curve (log rank test) showing OS for the predicted high-TIL and low-TIL subtypes in the TCGA test cohort. (**E**) Receiver operating characteristic (ROC) curves of predictions based on 11 gene expression levels in ACRG validation cohort, *n* = 295. (**F**) Kaplan–Meier curve (log rank test) showing OS for the predicted high-TIL and low-TIL in the ACRG validation cohort.

Although the high-TIL subtype showed a significantly different survival advantage, there was no quantitative method to determine which cluster an individual patient belonged to. Thus, a scoring system to accurately predict the molecular subtype in an individual patient is necessary. Firstly, the overlapping DEGs identified by DESeq2 and EdgeR between the high-TIL and low-TIL were submitted to the STRING website to obtain a comprehensive protein–protein interaction (PPI) network, and a total of 16 hub genes (*PRF1, GNLY*, *IL2RB*, *NKG7*, *GZMB*, *CXCL11*, *CXCR3*, *CCL5*, *CD8A*, *GZMA*, *GBP5*, *IDO1*, *ACTG2*, *CNN1*, *GBP1* and *MYH11*) were identified by cytoscape plug cytoHubba based on the PPI network (Figure 6A). Using the R package glmnet [31], a penalized multiple logistic regression on all hub genes simultaneously was performed. A total of 11 powerful predictive genes (*NKG7*, *GZMB*, *IL2RB*, *CCL5*, *CD8A*, *IDO1*, *MYH1*, *GNLY*, *CXCL11*, *GBP5* and *PRF1*; Figure 6B) were selected for the final scoring formula as follows.

Prediction-Score = 0.733 × *NKG7* + 0.566 × *GZMB* + (−0.434) × *IL2RB* + 0.379 × *CCL5* + 0.266 × *CD8A* + 0.122 × *IDO1* + (−0.245) × *MYH1* + 0.202 × *GNLY* + 0.153 × *CXCL11* + (−0.145) × *GBP5* + (−0.004) × *PRF1* − 7.78

Lastly, the performance of the Prediction-Score in predicting subtypes of GC patients was evaluated. Based on the Prediction-Scores from training dataset, samples with a Prediction-Score greater than 0.276 were regarded as a high-TIL subtype. According to the same analysis and standard, the model achieved significant sensitivity and accuracy on the TCGA test dataset comprising 84 STAD patients and the AUC value reached 0.945 (Figure 6C). We further tested the model on the ACRG dataset, and the AUC values still reached 0.814 even with genetic heterogeneity existing between the two cohorts (Figure 6E). Based on the predicted subtypes on both TCGA test dataset and ACRG dataset, patients with higher Prediction-Scores (predicted high-TIL subtype) exhibited significant survival advantage than patients with lower Prediction-Scores (predicted low-TIL subtype) (Figure 6D,F). However, the result is statistically significant with 94.1% confidence due to the smaller sample size in the test set, which is less than the typical 95% confidence. These results demonstrated that the predictive model effectively stratified GC patients based on the personalized genomic data.

## 4. Discussion

Cancer immunotherapy has been one of the most promising therapies for cancer treatment in recent years [37]. This approach is employed in the management of many gastric cancer patients [38], and considerable progress is being made in devising new immunotherapeutic strategies for GC. In this study, we explored immune cell composition in gastric cancer tumors by revealing the extent of tumor-infiltrating lymphocytes and its response to immune checkpoint inhibitors (ICIs).

We initially evaluated 22 immune cell types in each tumor samples and only kept credible samples with statistical significance (*p* < 0.05), which contains 303 tumor samples and 19 normal samples. By conducting comparative analysis on immune cells between tumor and normal samples, more cells such as Macrophage M0, M1, M2, Tregs and activated CD4 memory T cells were observed to be present in the tumor samples. These results showed that the correlations among immune cells in tumor and normal samples are quite different. We further classified all samples into high-TIL and low-TIL subtypes based on prognosis and immune cell infiltration in 282 cases with both survival information and gene expression data. The 5-year survival rate of high-TIL was kept stable at 58% since the 2.5th year, but it was lower than 30% in low-TIL, showing that tumors had high immunity infiltration could have a better prognosis. CD8 T cells, activated CD4 memory T cells, follicular helper T cells and Macrophage M1 were highly infiltrated in the high-TIL subtype. Cytotoxic CD8+ T cells (CTLs) are a major population of immune cells that control and clear tumor cells [39]. Activated CD4 memory T cells enhance CD8 T cells responses [40,41,42]. Follicular helper T cells regulate antigen-specific B cell immunity [43]. M1 macrophages participate in the positive immune response and function as an immune monitor [44]. These types of cells will promote the presentation of tumor antigens and finally kill tumor cells. Compared with the TCGA molecular classification of tumor samples, we found that either high-TIL or low-TIL subtype comprised heterogenous TCGA subtypes. Notably, the 72.7% EBV and 42.9% MSI subtypes belong to the high-TIL group, while 88.6% GS and 84.8% CIN samples were classified into the low-TIL subtype. Thus, the TIL classification based on the immune scores provides additional information for patient stratification that is independent from the TCGA molecular classification.

Our DEG analysis identified 189 up-regulated genes and 995 down-regulated genes in the high-TIL subtype. Both GO and KEGG analyses revealed that immune-related pathways were enriched in up-regulated genes (Figure 4C,D). These results indicated that the tumors of high-TIL subtype have a broadly elevated immune response. EBV-positive gastric cancer frequently has *PD-L1* amplification [45], and we also found that the expressions for both *PD-1* and ligand *PD-L1* (Figure 4B) are significantly higher in high-TIL subtypes. Similarly, mutation analysis showed that TMB and the counts of mutated genes in the high-TIL subtype are significantly higher than that in the low-TIL subtype. High *PD-L1* expression and high TMB are both important biomarkers for response to ICIs [36]. Thus, it is a reasonable hypothesis that the residual high-TIL tumor cells would easily be recognized and killed by the patient immune system, which might explain why the survival rate is stable after 2.5 years in high-TIL patients.

To ascertain whether a patient belongs to the high-TIL subtype, we constructed a PPI network with DEGs and then built a shrink network containing 16 hub genes that were identified by the cytoscape plug cytoHubba based on the network. We used an elastic net to further shrink the variables and built a predicting model with 11 genes (*NKG7*, *GZMB*, *IL2RB*, *CCL5*, *CD8A*, *IDO1*, *MYH1*, *GNLY*, *CXCL11*, *GBP5* and *PRF1*). The predictive AUCs on the test dataset and validation set showed the effectiveness of this model. Notably, all eleven genes are immune-related genes, with the exception of *MYH11*. For example, *IDO1* is the most represented in DEG analysis (Figure 4B). Most human tumors have *IDO1* expression, which helps induce disease immune tolerance [46]. *NKG7* expressed by natural killer cells was critical for controlling cancer initiation [47], growth and metastasis. The study has revealed new insights into immunotherapy in gastric cancer.

## Data Availability

The data supporting the findings of this study are included within the article.

## Supplementary Materials

The following supporting information can be downloaded at: https://www.mdpi.com/article/10.3390/genes13061017/s1. Figure S1: Identification of optimal cluster for tumor samples; Figure S2: The distribution of (A) sex, (B) age, (C) Lauren classification and (D) TCGA molecular classification in high-TIL and low-TIL.
