# Supplementary material for: Profiling the Tumor-Infiltrating Lymphocytes in Gastric Cancer Reveals Its Implication in the Prognosis
Source: Genes (Basel). 2022 Jun 5;13(6):1017. doi: 10.3390/genes13061017 (PMC9222794; doi:10.3390/genes13061017)
Supplement: Supplementary file 1 [file genes-13-01017-s001.zip › genes-1746852-supplementary.pdf]

Supplementary figure legends

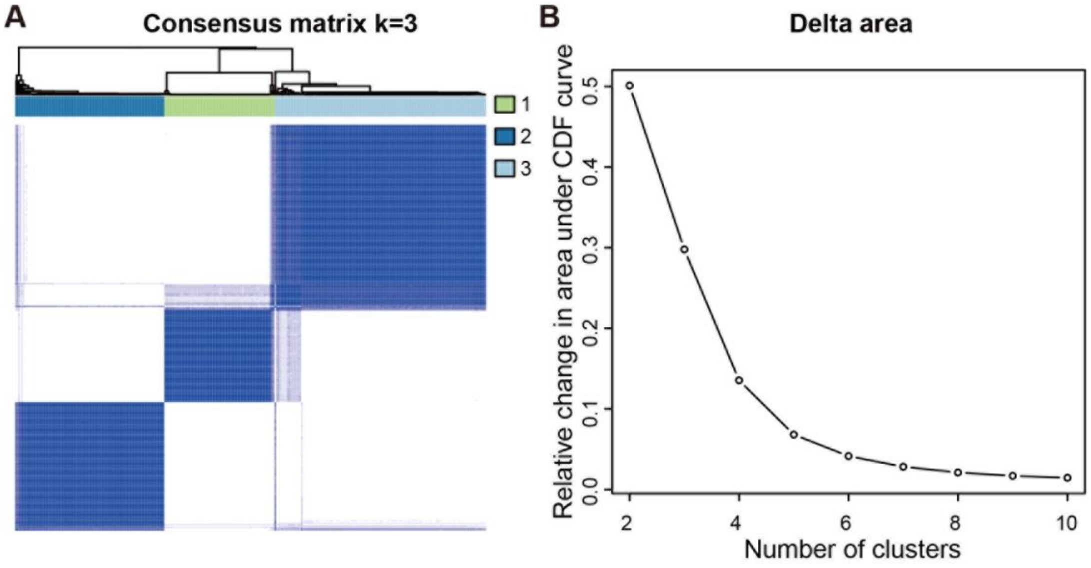

Figure S1. Identification of optimal cluster for tumor samples. (**A**) Clustered heatmap showed the clusters of samples based on immune cells composition. (**B**) Delta diagram showed the clusters with under area.

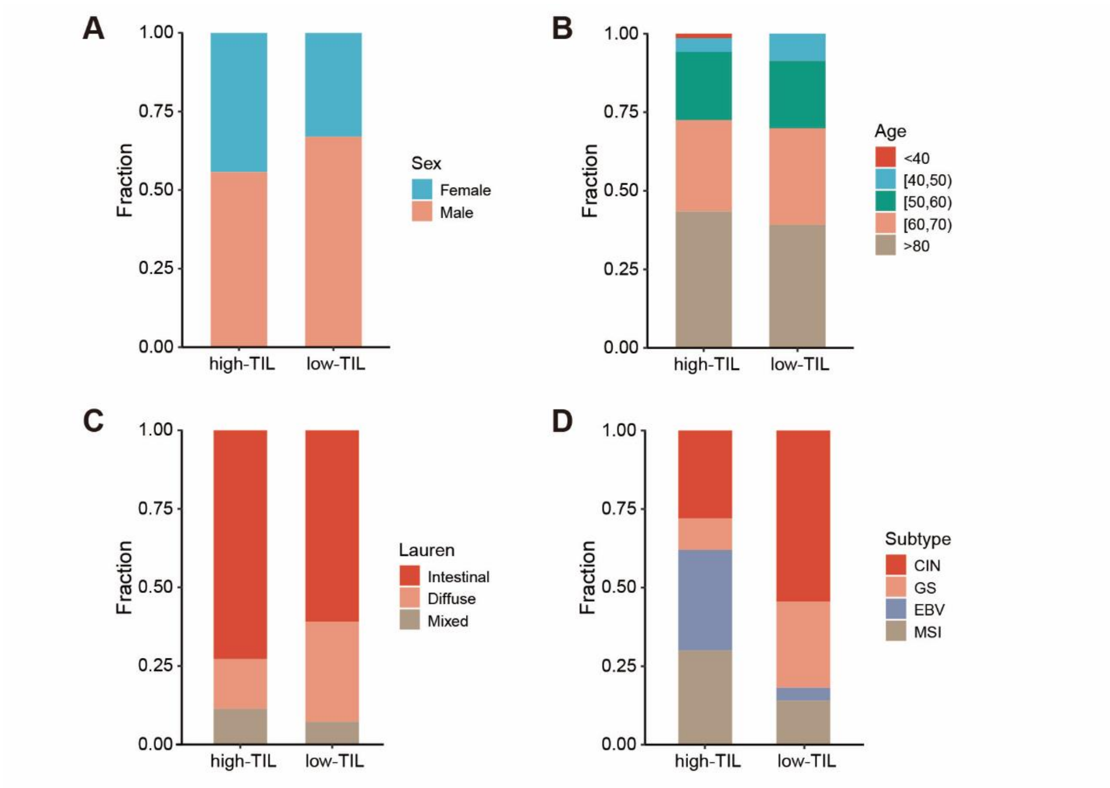

Figure S2. The distribution of (**A**) sex, (**B**) age (**C**) Lauren classification, and (**D**) TCGA molecular classification in high-TIL and low-TIL.
